# Supplementary material for: Horizontal Integration and Financing Reform of Rural Primary Care in China: A Model for Low-Resource and Remote Settings
Source: Int J Environ Res Public Health. 2022 Jul 8;19(14):8356. doi: 10.3390/ijerph19148356 (PMC9323543; doi:10.3390/ijerph19148356)
Supplement: Supplementary file 1 [file ijerph-19-08356-s001.zip › ijerph-1740215-supplementary.pdf]

Supplementary Material:

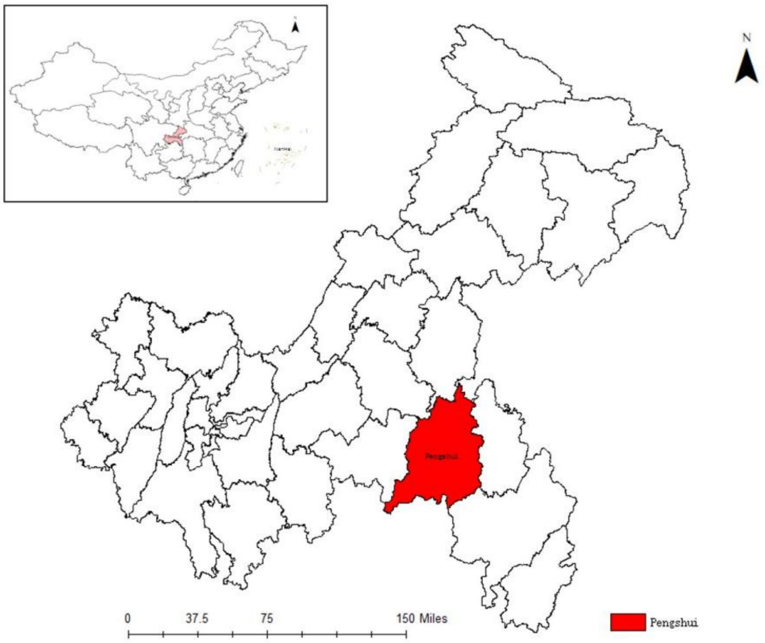

Figure S1. The location of Pengshui County, Chongqing, China.

Supplementary Material:

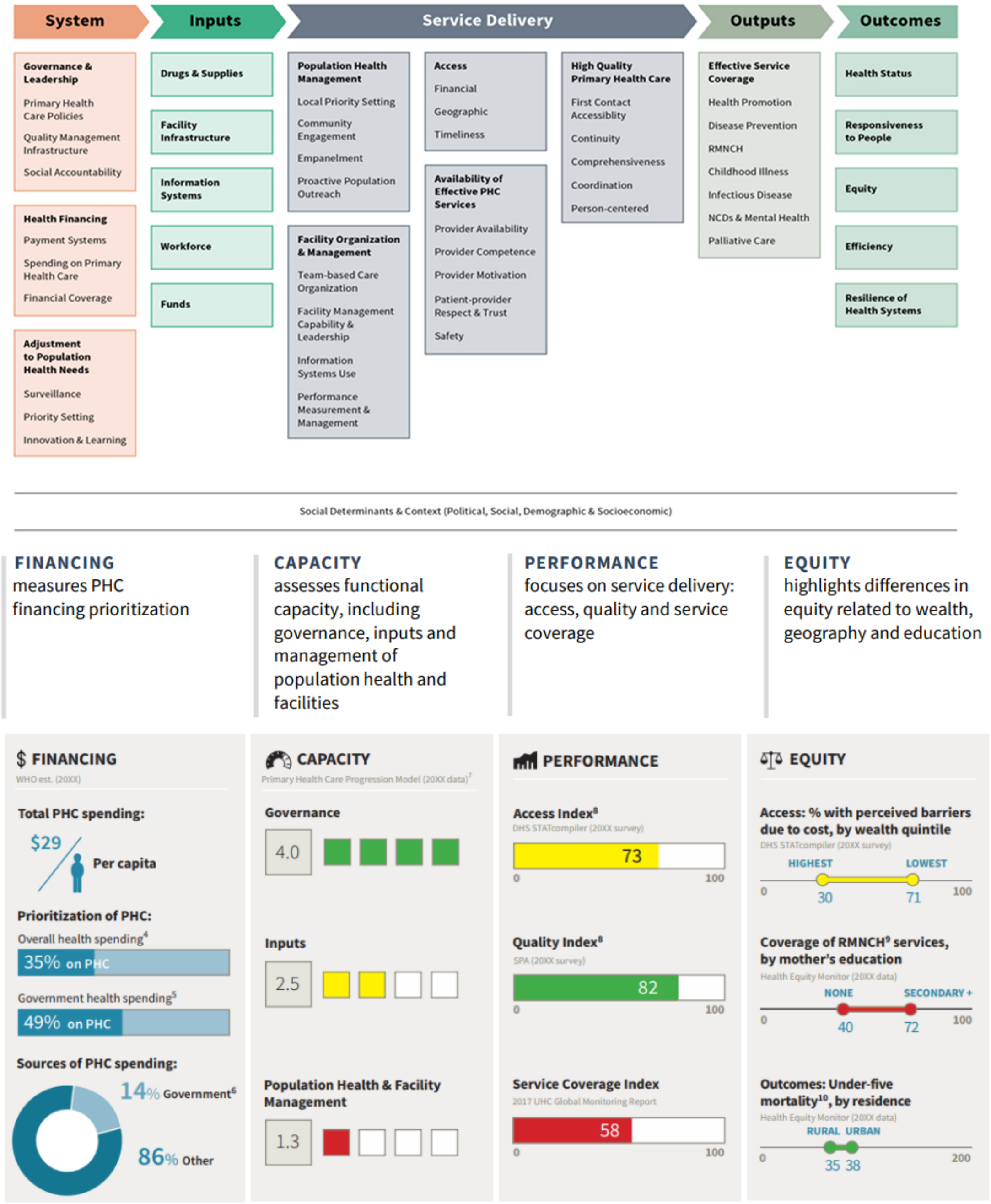

Figure S2. The Primary Health Care Performance Initiative conceptual framework and PHC Vital Signs Profiles [30,31]
